# Optoelectronic tuning of plasmon resonances via optically modulated hot electrons

**DOI:** 10.1093/nsr/nwad280

**Published:** 2023-11-03

**Authors:** Jiacheng Yao, Cheng Wang, Chi Zhang, Song Ma, Li Zhou, Ti Wang, Ququan Wang, Hongxing Xu, Tao Ding

**Affiliations:** Key Laboratory of Artificial Micro/Nano Structure of Ministry of Education, School of Physics and Technology, Wuhan University, Wuhan 430072, China; Key Laboratory of Artificial Micro/Nano Structure of Ministry of Education, School of Physics and Technology, Wuhan University, Wuhan 430072, China; Key Laboratory of Artificial Micro/Nano Structure of Ministry of Education, School of Physics and Technology, Wuhan University, Wuhan 430072, China; Key Laboratory of Artificial Micro/Nano Structure of Ministry of Education, School of Physics and Technology, Wuhan University, Wuhan 430072, China; Key Laboratory of Artificial Micro/Nano Structure of Ministry of Education, School of Physics and Technology, Wuhan University, Wuhan 430072, China; Key Laboratory of Artificial Micro/Nano Structure of Ministry of Education, School of Physics and Technology, Wuhan University, Wuhan 430072, China; Department of Physics, Southern University of Science and Technology, Shenzhen 518055, China; Key Laboratory of Artificial Micro/Nano Structure of Ministry of Education, School of Physics and Technology, Wuhan University, Wuhan 430072, China; Key Laboratory of Artificial Micro/Nano Structure of Ministry of Education, School of Physics and Technology, Wuhan University, Wuhan 430072, China

**Keywords:** hot carriers, plasmon switching, optoplasmonics, circuit model, NPoM

## Abstract

Fast optical modulation of nanoplasmonics is fundamental for on-chip integration of all-optical devices. Although various strategies have been proposed for dynamic modulation of surface plasmons, critical issues of device compatibility and extremely low efficiency in the visible spectrum hamper the application of optoplasmonic nanochips. Here we establish an optoplasmonic system based on Au@Cu_2__–__x_S hybrid core–shell nanoparticles. The optical excitation of hot electrons and their charge transfer to the semiconductor coating (Cu_2__–__x_S) lead to lowered electron density of Au, which results in the red shift of the localized surface plasmon resonance. The hot electrons can also transport through the Cu_2__–__x_S layer to the metal substrate, which increases the conductance of the nanogap. As such, the coupled gap plasmon blue-shifts with a magnitude of up to ∼15 nm, depending on the excitation power and the thickness of the coatings, which agrees with numerical simulations. All of this optoelectronic tuning process is highly reversible, controllable and fast with a modulated laser beam, which is highly compatible and sufficiently useful for on-chip integration of nanophotonic devices.

## Teaser

Optically excited hot electrons modulate the plasmon resonances via ultrafast charge transfer, which is highly compatible for optoplasmonic devices and photonic chip integration.

## INTRODUCTION

Nanoplasmonic devices have been regarded as the fundamental component for the next generation of information technology due to its ultrafast speed, ultracompact size and extremely low heat dissipation [1]. Although this concept was brought in about a decade ago, the bottleneck issues of modulation speed and depth as well as compatibility with existing optoelectronic technology have not been satisfactorily resolved. For instance, although diverse plasmon switching strategies have been developed, most of them are slow in speed unless optical or optoelectronic means are applied [2]. Thus, optical modulation of surface plasmons based on the pump-probe technique has been developed but high pump power is required for an appreciable change [3–5]. Other optical switching strategies based on photothermal effect [6–10] or optoelectronic effect [11–14] are either not device-compatible or only suitable for the middle infrared (MIR) or near infrared (NIR) region, which does not align well with the theme of optical communication and computation.

Direct modulation of the carrier density of the plasmonic components with light is possible but the efficiency is low unless a quantum tunneling region is reached [15]. It is critical that the conductance of the gap materials can be modulated by light in the order of dozens of G_0_ (quantum conductance) [16], which is not applicable for most inorganic/organic materials [17]. Perhaps the ideal material for ultrafast plasmon switching is vanadium dioxide with Mott transition [18,19], which, however, is challenging to fit into plasmonic hotspots with a thickness of a few nanometers [20–22].

Cu_2__–__x_S is a narrow-bandgap material that has shown strong capability of charge transfer (CT) for plasmon tuning [23]. However, this tuning strategy is slow and not reversible as it is based on wet chemistry, which makes it notoriously incompatible for device-based applications. Here we attempt to optically modulate the plasmon resonances of Au@Cu_2__–__x_S core–shell nanoparticles (NPs), which show reversible red shift of localized surface plasmon resonance (LSPR) due to the deficiency in electron density on Au caused by the CT process. In contrast, when these Au@Cu_2__–__x_S NPs are placed on Au films, conductance of the nanogaps is boosted by this CT, which results in reversible blue shift of the coupled plasmons. Both the experimental data and numerical analysis consistently support the proposed mechanism of optoelectronic tuning of surface plasmons, which is based on the optical attenuation of the plasmonic hot electrons. This type of tuning mechanism offers the possibility of fast plasmonic switching with light programmability and remote controllability, which are highly compatible for optoplasmonic devices.

## RESULTS AND DISCUSSION

**Figure 1.**
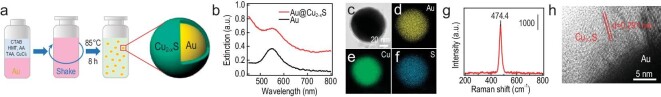
Synthesis of Au@Cu_2__–__x_S core–shell NPs and their characterizations. (a) Synthetic procedure of Au@Cu_2__–__x_S core–shell NPs. (b) Extinction spectra of Au and Au@Cu_2__–__x_S core–shell NPs. (c) TEM and (d)–(f) elemental mapping of the Au@Cu_2__–__x_ NPs. (g) Raman spectrum of Au@Cu_2__–__x_S NPs. (h) HRTEM image of a typical Au@Cu_2__–__x_S core–shell NP.

**Figure 2.**
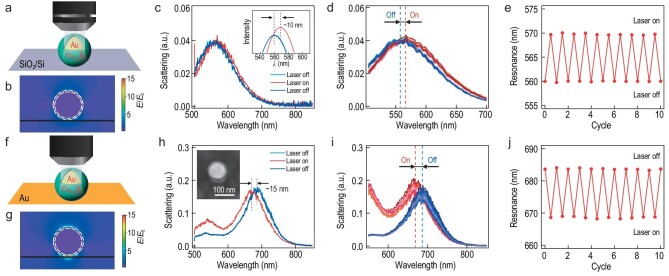
Laser-induced plasmon switching of Au@Cu_2__–__x_S NPs on (a)–(e) SiO_2_/Si substrate and (f)–(j) Au substrate. (a) and (f) Schematic of laser-induced switching; (b) and (g) electric near-field profile; (c) and (h) scattering spectra of Au@Cu_2__–__x_S with laser switched on and off; (d) and (i) scattering spectra of Au@Cu_2__–__x_S with cycles of laser on and off; and their (e) and (j) reversible resonance shifts with cycles of laser on and off.

The Au@Cu_2__–__x_S NPs were synthesized via sol–gel chemistry, where the Cu^2+^ ions are partially reduced by ascorbic acid (AA) and combine with S^2−^ ions disassociated from thioacetamide (TAA). As a result, the Cu_2__–__x_S NPs grow on the Au NPs via heterogeneous nucleation, forming Au@Cu_2__–__x_S core–shell NPs with variable shell thickness (Fig. 1a). The slight red shift (∼5 nm) of extinction spectra suggests the coating of the Cu_2__–__x_S shells due to the increase in the refractive index (RI) (Fig. 1b). Transmission electron microscope (TEM) imaging and energy dispersive X-ray spectroscopy (EDS) confirm that the shell has a thickness of ∼5 nm with elements of Cu and S (Fig. 1c–f). X-ray photoelectron spectroscopy (XPS) along with the Auger spectrum further confirms the presence of Cu(I) in the Au@Cu_2__–__x_S NPs (Supplementary Figure S1) [24]. Raman spectrum of the Au@Cu_2__–__x_S NPs presents a prominent peak at 474.4 cm^−1^, which is attributed to the stretching of the S–S bond [25]. High-resolution TEM (HRTEM) further reveals the crystalline feature of the Cu_2__–__x_S shells, which agrees with its lattice constant as reported in the literature [26]. The hybrid core–shell particles are normally dispersed in aqueous solution, which can be drop-casted on SiO_2_/Si wafer (Fig. 2a) or Au substrate (Fig. 2f) for light-induced plasmon switching.

For Au@Cu_2__–__x_S NP on SiO_2_/Si substrate, it shows weak electric (E)-field enhancement (∼5) with LSPR (Fig. 2b). Illumination from a continuous wave (CW) laser (446 nm) on the Au@Cu_2__–__x_S NP results in a ∼10-nm red shift of the scattering peak, which can switch back when the laser is turned off (Fig. 2c). This light-induced plasmon tuning is fully reversible for many cycles of light on and off (Fig. 2d and e), which excludes the possibility of irreversible particle deformation during the laser irradiation as shown previously [27]. On the contrary, the structure of Au@Cu_2__–__x_S NP on Au film with strong E-field enhancement (∼20, Fig. 2g) shows blue shift (∼15 nm) of the scattering peak with laser illumination, which can shift back when the laser is switched off (Fig. 2h). Note that photoluminescence (PL) signals of plasmons are also collected during the laser excitation [28,29], which are subtracted to reflect the real change in white light scattering (Supplementary Figure S2). This blue shift, again, is fully reversible for many cycles of laser on and off (Fig. 2i and j).

**Figure 3.**
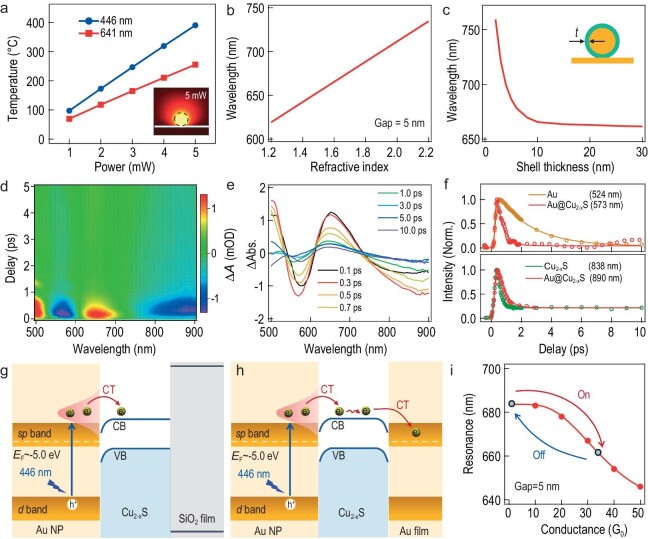
Mechanism investigation of CT in the Au@Cu_2__–__x_S NPoM system and its influence on the plasmon shift. (a) Temperature calculation of the Au NP irradiated with different laser powers. Inset is the temperature profile around Au with irradiation power of 5 mW. Change in simulated plasmon resonance with (b) RI of the gap medium and (c) the shell thickness. TA spectra of Au@Cu_2__–__x_S NPs: (d) Kinetic mapping of TA spectra, (e) TA spectra at different probe time, (f) comparison of the electron decay dynamics of Au NP, Au@Cu_2__–__x_S and Cu_2__–__x_S, respectively. Schematics of the CT processes in the system of Au@Cu_2__–__x_S NP on (g) SiO_2_/Si substrate and (h) Au substrate, respectively. (i) Calculated change in plasmon resonance with gap conductance.

This peculiar contrast suggests that some unique underlying mechanism is involved. As is well known, plasmon shift is normally induced by a change in RI, a change in separation and a change in charge density [2]. The former two are likely as the deformation of Cu_2__–__x_S or the change in RI induced by the photothermal effect of laser irradiation can result in the shift of plasmon as revealed previously [30,31]. This is a reasonable assumption as the local temperature can reach ≤400°C for both 446- and 641-nm laser irradiation (Fig. 3a), which is sufficient to cause the phase change of Cu_2__–__x_S [32]. However, we observe a slight blue shift (∼3 nm) or no shift with 641-nm laser irradiation for Au@Cu_2__–__x_S on SiO_2_/Si or Au substrate (Supplementary Figure S3a and b), suggesting that these shifts of plasmon resonance are not likely caused by the increase in temperature. Moreover, for the observed 15-nm blue shift, it theoretically requires a decrease of 0.2 in RI or an increase of ∼8 nm in gap size (Fig. 3b and c and Supplementary Figure S4), which, however, is not likely as the RI of Cu_2__–__x_S tends to increase and the thickness decreases due to the phase change [32,33]. Therefore, the only possibility it seems is the change in carrier density of the Au cores upon laser irradiation, which is highly related to the probability of hot electron generation and injection induced by the strong field enhancement of Au NPs and nanoparticles-on-mirror (NPoMs) (Fig. 2b and g). Hot electrons are normally generated with resonance excitation, which experience radiative and non-irradiative decay channels via electron–electron and electron–phonon scattering [34,35]. When a semiconductor (here the Cu_2__–__x_S) is in contact with Au NP, these hot electrons can enter the conduction band of the semiconductor, which reduces the electron density on the Au. For NPoM, the injected hot electrons can further transport to Au films forming an electric short pass, thereby increasing the gap conductance. Both effects can lead to the shift of plasmons.

The plasmon shift caused by the change in charge density can be analytically calculated as [36]:


(1) \begin{equation*}\Delta \lambda = - \frac{{\Delta N}}{{2N}}{\lambda }_{\mathrm{P}}\sqrt {{\varepsilon }_\infty + \left( {\frac{1}{P} - 1} \right){\varepsilon }_{\mathrm{d}}} ,\end{equation*}

where ${\varepsilon }_{\mathrm{d}}$ is the permittivity of the surrounding dielectric medium, ${\varepsilon }_\infty $ is the background high-frequency permittivity, *P* is the shape-dependent depolarization factor, ${\lambda }_{\mathrm{P}}$ is the plasmon wavelength, *N* is the carrier density and $\Delta N$ is the change in carrier density. The carrier density of Au cannot be easily modified by laser irradiation (446 nm) due to its high work function (5.0 eV). However, with the coating of semiconductor material such as the Cu_2__–__x_S, hot electron transfer from Au to Cu_2__–__x_S is possible. This is verified by transient absorption (TA) spectra, where the Au@Cu_2__–__x_S NPs show two typical modes at 573 and 890 nm, corresponding to the plasmon resonances of Au and Cu_2__–__x_S, respectively (Fig. 3d and e). The TA of Au@Cu_2__–__x_S NPs (at 573 nm) presents a lifetime of 0.38 ps, which is approximately six times faster than that of Au NPs (Fig. 3f upper panel and Supplementary Figure S5a and c), suggesting that CT happens from Au to the Cu_2__–__x_S shells. As the Cu_2__–__x_S shells receive electrons from Au, their TA spectra decay (at 890 nm) also appears slightly slower than that of the Cu_2__–__x_S NPs (Fig. 3f lower panel and Supplementary Figure S5b and d). As such, the electron density of Au is reduced due to such CT (Fig. 3g), which results in a positive value of Δ*λ* as predicted by using Equation (1). Thus, red shift of LSPR is observed when the excitation laser is on, which can shift back due to electron backflow when the laser is switched off (Fig. 2c). With 641-nm laser irradiation, however, no obvious electron dynamics was observed for pure Au NPs (Supplementary Figure S6a) but a clear CT process still happens in the hybrid Au@Cu_2__–__x_S NPs (Supplementary Figure S6b), indicating that 641-nm light mainly excites the Cu_2__–__x_S, whose electrons transfer to the Au NP (Supplementary Figs S3c and S6c and d). As such, the electron density of Au NP increases, which results in a slight blue shift (Supplementary Figure S3a) as predicted by using Equation (1).

When Au@Cu_2__–__x_S NPs are placed on metal films, NPoM constructs are established and a circuit model is applied to qualitatively analyse the antenna mode of this plasmonic system as [16]:


(2) \begin{equation*}\lambda = {\lambda }_{\mathrm{p}}\sqrt {{\varepsilon }_\infty + 2{\varepsilon }_{\mathrm{d}} + 4{\varepsilon }_{\mathrm{d}}{C}_{\mathrm{g}}/{C}_{{\mathrm{NP}}}} ,\end{equation*}

where ${C}_{\mathrm{g}}$ and ${C}_{{\mathrm{NP}}}$ are the capacitance of the structure and NP, respectively. Here we approximate ${C}_{\mathrm{g}}$ using the formula ${C}_{\mathrm{g}} = {\varepsilon }_0n_{\mathrm{g}}^2( {A/d} )$, where *A* and *d* are the area of the bottom facet of the Au NP and the gap size, respectively [37]. Since the capacitance is mainly determined by the size of the gap/NP and their dielectric properties, modifying either of these parameters will shift the plasmon resonances ($\lambda $). In a certain NPoM structure in which the gap size and dielectric environment are fixed, the gap conductance is critical to the shift in plasmon resonances. The conductance increase contributed from the photothermal effect is small (0.2G_0_, Supplementary Figure S7) [38]. Thus, the major contribution to the conductance increase is the hot electron transfer from Au to Cu_2__–__x_S, which then transports to the Au substrate (Fig. 3h). The conductivity of Cu_2__–__x_S in the nanogap can be calculated as${\mathrm{\ }}\sigma = n \cdot \mu \cdot e$, where *n* and $\mu $ are the charge density and carrier mobility within Cu_2__–__x_S. *e* is the unit charge. For 80 nm of Au NPoM with a facet diameter of 10 nm and gap of 5 nm, the calculated gap conductance increase due to hot electron tunneling is ∼32G_0_ (see Methods for the detailed calculation). This large conductance increase can lead to a 18-nm blue shift as predicted by using numerical analysis (Fig. 3i and Supplementary Figure S8), which agrees with the blue shift observed in the experiments (Fig. 2h). Again, with 641-nm laser excitation, because the CT process happens from Cu_2__–__x_S to Au NPs or Au films (Supplementary Figure S3d), a negligible change in gap conductance is expected, which reasonably explains no shift in the coupled plasmons (Supplementary Figure S3b).

**Figure 4.**
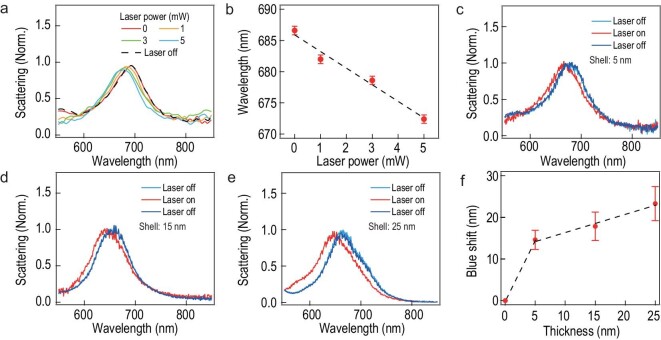
Power- and thickness-dependent plasmon shift of Au@Cu_2__–__x_S NPoMs. (a) Scattering spectra of Au@Cu_2__–__x_S NPoM at different irradiation power. (b) Change in plasmon resonance with irradiation power. (c)–(e) Scattering spectra of Au@Cu_2__–__x_S NPoMs of different shell thickness: (c) 5 nm, (d) 15 nm, (e) 25 nm. Pumping power: 5 mW. (f) Change in blue-shift magnitude with shell thickness. All the error bars are the averaged and squared deviation based on five NPoMs.

As the plasmon resonance is determined by the gap conductance, increasing the irradiation power would lead to an increase in the hot carrier concentration (*n*), which would increase the gap conductance (*G*). Therefore, we see an increasing blue shift of the gap plasmons with the increase in irradiation power (Fig. 4a and b). The magnitude of the blue shift can also be attenuated with shell thickness. Here, we synthesized Au@Cu_2__–__x_S NPs of different shell thicknesses by adding different amounts of precursors, which yields uniform shells ranging from 5 to 25 nm (Supplementary Figure S9). As the shell thickness increases, the magnitude of the blue shift increases proportionally at the same pumping power (Fig. 4c–f), which is likely due to the increased carrier mobility of thicker shells [39].

## CONCLUSION

In summary, we have demonstrated a dynamic and reversible tuning strategy of plasmon resonances based on optical modulation of hot carrier transport in the semiconductor coating. The hot electrons are generated from interband excitation (446 nm) of Au NP and transfer to the Cu_2__–__x_S coating, which reduces the electron density of Au, thereby red-shifting the LSPR. In the NPoM geometry, such CT enhances the conductance of the plasmonic nanogaps, which results in blue shift of the coupled plasmon resonances. Excitation with a longer-wavelength laser (641 nm) mainly results in excitation of Cu_2__–__x_S, which induces CT from Cu_2__–__x_S to Au. As such, we see a slight blue shift of the plasmons due to the increase in the electron density. But this CT does not strongly affect the coupled plasmons in the NPoM geometry as it does not significantly alter the gap conductance. A finite element method (FEM) is applied to simulate the conductance-induced plasmon shift, which agrees well with the experimental results. Unlike the conventional plasmon-tuning strategy, this intriguing tuning mechanism is based on optoelectronic excitation of hot carriers as well as the modification of electron density and gap conductance, which is fast (∼THz) and chip-compatible, meeting the high demands of the ultracompact on-chip integration of functional nanophotonic circuits and photonic computation.

## METHODS

### Synthesis of Au@Cu_2__–__x_S core–shell NPs

The Au@Cu_2__–__x_S core–shell NPs were synthesized according to a previous report [26]. Specifically, 50 μL of 0.2 M cetyltrimethylammonium bromide (CTAB), 50 μL of 0.1 M hexamethylenetetramine (HMT), 50 μL of 0.1 M AA, 50 μL of 0.01 M TAA and 0.01 M cupric chloride (CuCl_2_) with varied volume were sequentially added into vials containing 1 mL of Au NP dispersion (Nanopartz), which were incubated at 85°C for 8 h. The hybrid core–shell NPs were harvested by centrifugation at 8000 r/min for 10 min and redispersed in 1 mL of deionized water for stock use.

### Characterizations

UV–vis extinction spectra of the Au NPs and hybrid Au@Cu_2__–__x_S core–shell NPs were collected using an optofiber spectrometer (QE65000, Ocean Optics). The Raman spectra of Cu_2__–__x_S were obtained using a confocal Raman microscope (Renishaw InVia) equipped with a 532-nm laser. TEM images and energy dispersive EDS were captured at an accelerating voltage of 200 kV (JEOL JEM-F200). Scanning electron microscope (SEM) images were obtained using a Sigma Zeiss at an accelerating voltage of 10 kV. XPS (Thermo Fisher ESCALAB 250Xi) were performed using a monochromatic Al K Alpha (1 486.68 eV) X-ray source under an ultra-high vacuum (10^−7^ mbar). The X-ray high-tension (HT) was 15 kV and the measured spot size was 200 μm.

### Optical switching of the plasmon resonances based on Au@Cu_2__–__x_S NPoM system

To construct the NPoM system, Au@Cu_2__–__x_S NPs were drop-casted on Au films, which were then placed under a dark-field (DF) microscope (BX53M, Olympus). The scattering spectra of the NPs were recorded confocally through a 100 × DF objective (numerical aperture (NA): 0.8) that were coupled using a 50-μm optical fiber connected to a spectrometer (integration time: 800 ms, QEPro, Ocean Optics). DF scattering spectra were collected simultaneously when an excitation laser (CW) of 446 nm was on. The power of the CW laser was 5 mW and the duration of the laser on/off periods was 1 s each. The PL spectra of the same Au@Cu_2__–__x_S NPoMs were recorded, which were then used to subtract the scattering spectra to exclude the influence of PL on the shift of plasmons (Supplementary Figure S2). The scattering spectra of Au@Cu_2__–__x_S NP on SiO_2_ substrate were measured in the same way as a contrast experiment.

### Ultrafast TA spectroscopy

The TA spectra of the Au NPs, Cu_2__–__x_S NPs and Au@Cu_2__–__x_S NPs were collected using a commercial TA system (HARPIA, Light Conversion). Specifically, a femtosecond laser (repetition rate of 40 kHz, 1030-nm central wavelength and pulse duration of ∼120 fs, PHAROS, Light Conversion) was split into two beams. One beam went through optical parametric amplification (ORPHEUS twins, Light Conversion) to pump the Au@Cu_2__–__x_S NPs. The other beam went through the delay line, which yields the white light via sapphire as the probe beam. The pump and probe beams were focused on Au@Cu_2__–__x_S dispersion in a cuvette with wavelengths of 400 and 640 nm. Single exponential fitting $y = {y}_0 + {a}_1 \cdot {e}^{\frac{{ - ( {t - {t_0} } )}}{\tau }}$ was applied to the TA spectra, which resolves the lifetime of *τ*.

### Simulations

The near-field scattering and temperature profile of Au@Cu_2__–__x_S NPs on Au film and SiO_2_ substrate are numerically simulated using FEM. Specifically, a plain wave incidence with energy density equivalent to a Gaussian beam was applied at the Au@Cu_2__–__x_S NPoMs. The thermal conductivity of Au, Cu_2__–__x_S and SiO_2_ are 318, 1.5 and 1.8 W (m·K)^−1^, respectively. The RI of Cu_2__–__x_S is set at an average value of 1.8 with an imaginary part of 0.02 [40,41]. Gap conductance is calculated based on the formula $G = \sigma \cdot \frac{S}{l}$, where *l* is the gap size (∼5 nm) and *S* is the area of the bottom facet of the Au NP with a radius of ∼5 nm. Conductivity of the gap was calculated based on the formula $\sigma = n \cdot \mu \cdot e$, where *n* is the carrier density change induced by the hot electron injection, which is ∼10^26^ m^−3^; $\mu $ is the carrier mobility of Cu_2_S, which is estimated to be 0.01 m^2^·(V·s)^−1^ [39]; and *e* is the unit charge (1.6 × 10^−19^).

## Supplementary Material

nwad280_Supplemental_File

## References

[1] Brongersma ML, Shalaev VM. The case for plasmonics. Science 2010; 328: 440–1. 10.1126/science.1186905 20413483

[2] Jiang N, Zhuo X, Wang J. Active plasmonics: principles, structures, and applications. Chem Rev 2018; 118: 3054–99. 10.1021/acs.chemrev.7b00252 28960067

[3] Wurtz GA, Pollard R, Hendren W et al. Designed ultrafast optical nonlinearity in a plasmonic nanorod metamaterial enhanced by nonlocality. Nat Nanotechnol 2011; 6: 107–11. 10.1038/nnano.2010.278 21258335

[4] Wang X, Morea R, Gonzalo J et al. Coupling localized plasmonic and photonic modes tailors and boosts ultrafast light modulation by gold nanoparticles. Nano Lett 2015; 15: 2633–9. 10.1021/acs.nanolett.5b00226 25798896

[5] Rotenberg N, Caspers JN, van Driel HM. Tunable ultrafast control of plasmonic coupling to gold films. Phys Rev B 2009; 80: 245420. 10.1103/PhysRevB.80.245420

[6] Chen F, Wang Y, Wang S et al. Plasmon-assisted nanopoling of poly(vinyl difluoride) films. Adv Opt Mater 2021; 9: 2100084. 10.1002/adom.202100084

[7] Cormier S, Ding T, Turek V et al. Actuating single nano-oscillators with light. Adv Opt Mater 2018; 6: 1701281. 10.1002/adom.201701281

[8] Li Q, Chen L, Xu H et al. Photothermal modulation of propagating surface plasmons on silver nanowires. ACS Photonics 2019; 6: 2133–40. 10.1021/acsphotonics.9b00711

[9] Kaya S, Weeber JC, Zacharatos F et al. Photo-thermal modulation of surface plasmon polariton propagation at telecommunication wavelengths. Opt Express 2013; 21: 22269–84. 10.1364/OE.21.022269 24104119

[10] Wang Y, Ding T. Optical tuning of plasmon-enhanced photoluminescence. Nanoscale 2019; 11: 10589–94. 10.1039/C9NR03725J 31120082

[11] Xing J, Zhao C, Zou Y et al. Modulating the optical and electrical properties of MAPbBr_3_ single crystals via voltage regulation engineering and application in memristors. Light Sci Appl 2020; 9: 111. 10.1038/s41377-020-00349-w 32637078 PMC7327067

[12] Michel A-KU, Chigrin DN, Maß TWW et al. Using low-loss phase-change materials for mid-infrared antenna resonance tuning. Nano Lett 2013; 13: 3470–5. 10.1021/nl4006194 23742151

[13] Rudé M, Simpson RE, Quidant R et al. Active control of surface plasmon waveguides with a phase change material. ACS Photonics 2015; 2: 669–74. 10.1021/acsphotonics.5b00050

[14] Seo M, Kyoung J, Park H et al. Active Terahertz nanoantennas based on VO_2_ phase transition. Nano Lett 2010; 10: 2064–8. 10.1021/nl1002153 20469898

[15] Zhang C, Li D, Zhang G et al. Switching plasmonic nanogaps between classical and quantum regimes with supramolecular interactions. Sci Adv 2022; 8: eabj9752. 10.1126/sciadv.abj9752 35119919 PMC8816333

[16] Benz F, de Nijs B, Tserkezis C et al. Generalized circuit model for coupled plasmonic systems. Opt Express 2015; 23: 33255–69. 10.1364/OE.23.033255 26831992

[17] King PDC, Veal TD. Conductivity in transparent oxide semiconductors. J Phys: Condens Matt 2011; 23: 334214. 10.1088/0953-8984/23/33/334214 21813954

[18] Qazilbash MM, Brehm M, Chae BG et al. Mott transition in VO_2_ revealed by infrared spectroscopy and nano-imaging. Science 2007; 318: 1750–3. 10.1126/science.1150124 18079396

[19] Rini M, Cavalleri A, Schoenlein RW et al. Photoinduced phase transition in VO_2_ nanocrystals: ultrafast control of surface-plasmon resonance. Opt Lett 2005; 30: 558–60. 10.1364/OL.30.000558 15789735

[20] Lei DY, Appavoo K, Sonnefraud Y et al. Single-particle plasmon resonance spectroscopy of phase transition in vanadium dioxide. Opt Lett 2010; 35: 3988–90. 10.1364/OL.35.003988 21124588

[21] Orlianges JC, Leroy J, Crunteanu A et al. Electrical and optical properties of vanadium dioxide containing gold nanoparticles deposited by pulsed laser deposition. Appl Phys Lett 2012; 101: 133102. 10.1063/1.4754708

[22] Boyce AM, Stewart JW, Avila J et al. Actively tunable metasurfaces via plasmonic nanogap cavities with Sub-10-nm VO_2_ films. Nano Lett 2022; 22: 3525–31. 10.1021/acs.nanolett.1c04175 35472261 PMC9101075

[23] Ma S, Yang DJ, Ding SJ et al. Tunable size dependence of quantum plasmon of charged gold nanoparticles. Phys Rev Lett 2021; 126: 173902. 10.1103/PhysRevLett.126.173902 33988417

[24] Majeski MW, Bolotin IL, Hanley L. Cluster beam deposition of Cu_2–__x_S nanoparticles into organic thin films. ACS Appl Mater Interfaces 2014; 6: 12901–8. 10.1021/am5028428 24977326

[25] Gómez-Solano RE, Arias-Cerón JS, Ríos-Ramírez JJ et al. Synthesis and study of structure and phase composition in Cu_2–__x_S, Sn_x_S_y_, ZnS, Cu_x_SnS_y_ and CuZnSnS pellets. J Mater Sci: Mater El 2020; 31: 7519–23.

[26] Ma S, Chen K, Qiu YH et al. Controlled growth of CdS–Cu_2−x_S lateral heteroshells on Au nanoparticles with improved photocatalytic activity and photothermal efficiency. J Mater Chem A 2019; 7: 3408–14. 10.1039/C8TA11154E

[27] Wang S, Ding T. Photothermal-assisted optical stretching of gold nanoparticles. ACS Nano 2019; 13: 32–7. 10.1021/acsnano.8b06087 30403333

[28] Li G-C, Zhang Y-L, Jiang J et al. Metal-substrate-mediated plasmon hybridization in a nanoparticle dimer for photoluminescence line-width shrinking and intensity enhancement. ACS Nano 2017; 11: 3067–80. 10.1021/acsnano.7b00048 28291332

[29] Zhang Q, Liu D, Ren Q et al. Probing electron transport in plasmonic molecular junctions with two-photon luminescence spectroscopy. Nanophotonics 2021; 10: 2467–79. 10.1515/nanoph-2021-0116

[30] Chen F, Liu Y, Ding T. Fast and hydrosensitive switching of plasmonic nanocavities via photothermal effect. Photon Res 2023; 11: 12–9. 10.1364/PRJ.470930

[31] Chen F, Yao J, Wang X et al. Fast modulation of surface plasmons based on the photothermal effect of nonvolatile solid thin films. Nanoscale 2023; 15: 476–82. 10.1039/D2NR05527A 36514986

[32] Wang S, Guo L, Wen X et al. Phase transitions in the Cu_2_S nanowires. Mater Chem Phys 2002; 75: 32–8. 10.1016/S0254-0584(02)00026-3

[33] Liang X, Jin D, Dai F. Phase transition engineering of Cu_2_S to widen the temperature window of improved thermoelectric performance. Adv Electron Mater 2019; 5: 1900486. 10.1002/aelm.201900486

[34] Ho KHW, Shang A, Shi F et al. Plasmonic Au/TiO_2_-dumbbell-on-film nanocavities for high-efficiency hot-carrier generation and extraction. Adv Funct Materi 2018; 28: 1800383. 10.1002/adfm.201800383

[35] Peng Z, Lo TW, Lei D. Plasmonic-hot-electron mediated room-temperature generation of charged biexciton in monolayer WS_2_. Phys Rev Materi 2023; 7: 054002. 10.1103/PhysRevMaterials.7.054002

[36] Mulvaney P, Pérez-Juste J, Giersig M et al. Drastic surface plasmon mode shifts in gold nanorods due to electron charging. Plasmonics 2006; 1: 61–6. 10.1007/s11468-005-9005-0

[37] Benz F, Tserkezis C, Herrmann LO et al. Nanooptics of molecular-shunted plasmonic nanojunctions. Nano Lett 2015; 15: 669–74. 10.1021/nl5041786 25494169 PMC4312133

[38] Bekenstein Y, Elimelech O, Vinokurov K et al. Charge transport in Cu_2_S nanocrystals arrays: effects of crystallite size and ligand length. Z Phys Chem 2015; 229: 179–90. 10.1515/zpch-2014-0593

[39] Ubale AU, Choudhari DM, Kantale JS et al. Synthesis of nanostructured Cu_x_S thin films by chemical route at room temperature and investigation of their size dependent physical properties. J Alloys Compd 2011; 509: 9249–54. 10.1016/j.jallcom.2011.07.009

[40] Zhang J, Xing T, Zhang M et al. Facile preparation of Cu_2-x_S supernanoparticles with an unambiguous SERS enhancement mechanism. Chem Eng J 2022; 434: 134457. 10.1016/j.cej.2021.134457

[41] Saadeldin M, Soliman HS, Ali HAM et al. Optical and electrical characterizations of nanoparticle Cu_2_S thin films. Chin Phys B 2014; 23: 046803. 10.1088/1674-1056/23/4/046803

