# Supplementary material for: Optoelectronic tuning of plasmon resonances via optically modulated hot electrons
Source: Natl Sci Rev. 2023 Nov 3;11(5):nwad280. doi: 10.1093/nsr/nwad280 (PMC10989291; doi:10.1093/nsr/nwad280)
Supplement: nwad280_Supplemental_File [file nwad280_supplemental_file.pdf]

Supplementary Materials

Optoelectronic tuning of plasmon resonances via optically modulated hot electrons

Jiacheng Yao[1], Cheng Wang[1], Chi Zhang [1], Song Ma [1], Li Zhou [1], Ti Wang [1], Ququan Wang[2], Hongxing Xu[1] and Tao Ding[1,*]

[1]Key Laboratory of Artificial Micro/Nano Structure of Ministry of Education, School of Physics and Technology, Wuhan University, Wuhan, 430072, China

[2]Department of Physics, Southern University of Science and Technology, Shenzhen, 518055, China

[*] **Corresponding author.** E-mail: t.ding@whu.edu

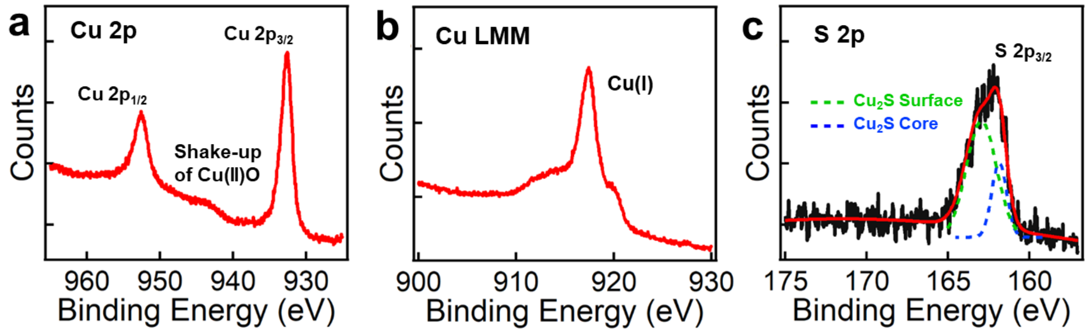

Figure S1 (a) Cu 2p, (b) Cu LMM and (c) S 2p XPS spectra for Au@Cu_{2-x}S NPs.

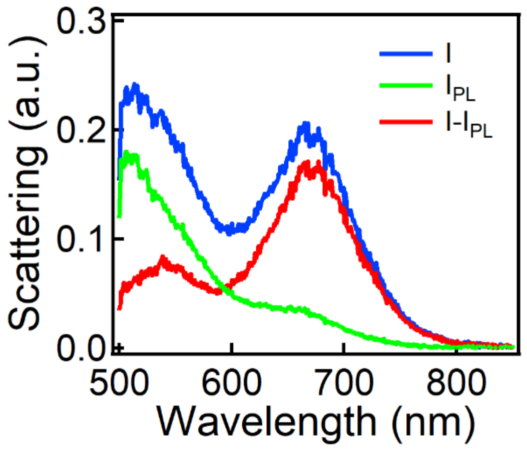

Figure S2 Laser excited scattering spectra of Au@Cu_{2-x}S NPoM with lamp on (blue line) and off (green line) and their subtraction (red line).

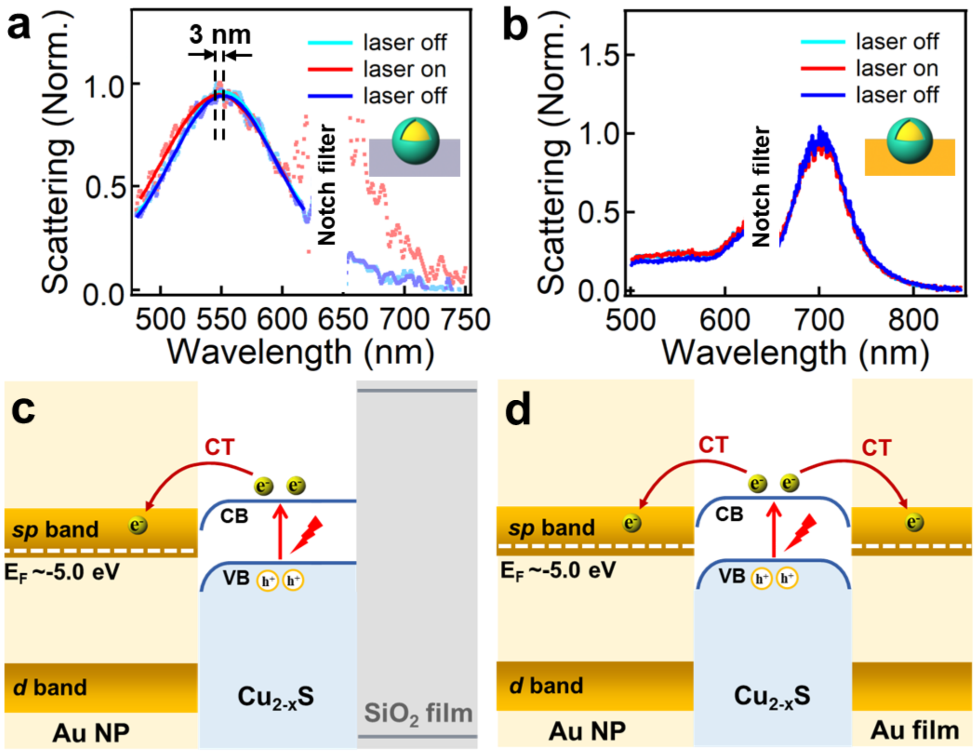

Figure S3 Wavelength-dependent plasmon tuning of Au@$Cu_{2-x}S$ NPs on different substrates. Scattering spectra of Au@$Cu_{2-x}S$ NPs on (a) SiO_2/Si and (b) Au substrates with laser (641 nm) switched on and off. Insets are schematic of the structure. Proposed charge transfer mechanism with the excitation of 641 nm laser for Au@$Cu_{2-x}S$ NPs on (c) SiO_2/Si and (d) Au substrates, respectively.

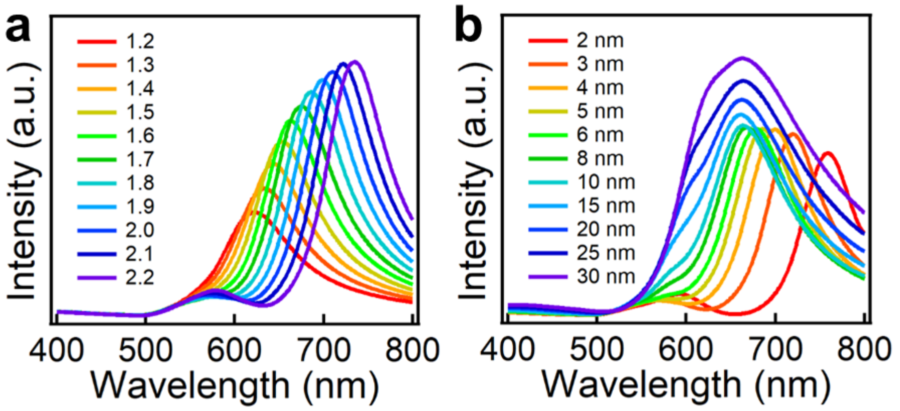

Figure S4 Simulated scattering spectra of Au@$Cu_{2-x}S$ NPoM with (a) different gap RI and (b) shell thickness.

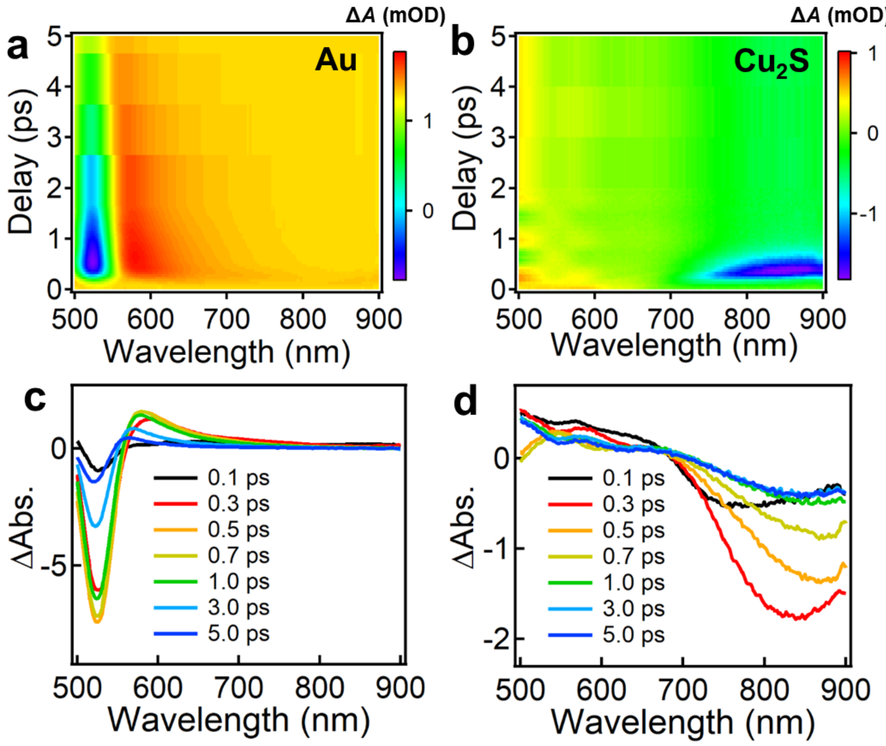

Figure S5 Transient absorption (TA) spectra of Au (a, c) NPs and (b, d) $Cu_{2-x}S$ NPs. (a, b) Kinetic mapping of TA spectra, and (c, d) TA spectra at different probe time. The pump laser is 400 nm and the probe beam is white light.

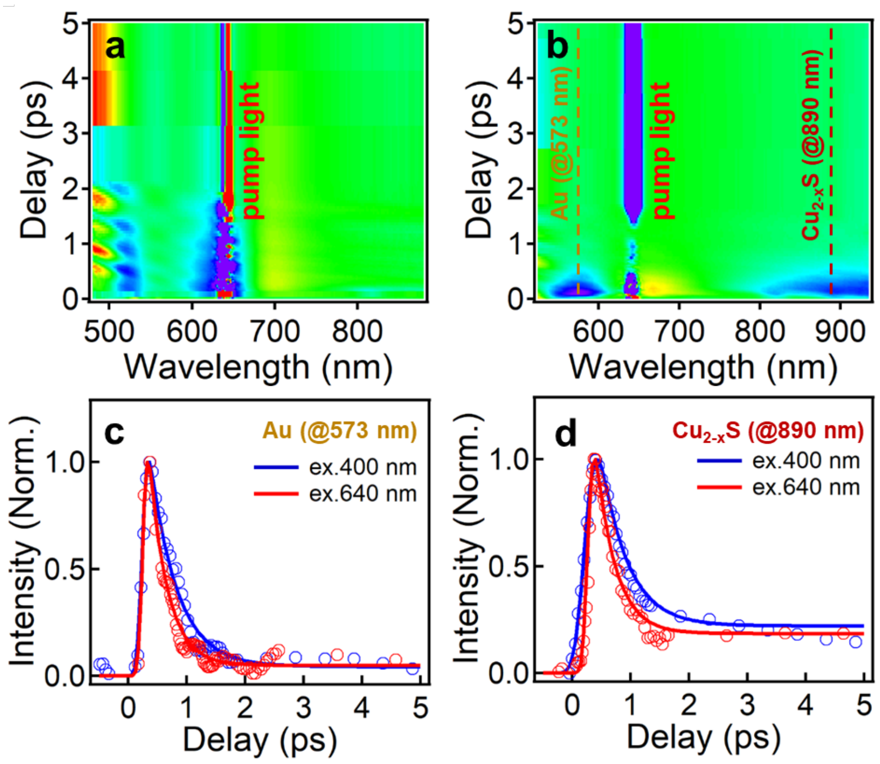

Figure S6 Transient absorption (TA) spectra of Au NPs and Au@$Cu_{2-x}S$ NPs with excitation of 640 nm pump laser. (a) Kinetic TA spectra mapping of Au NPs, (b) Kinetic TA spectra mapping of Au@$Cu_{2-x}S$ NPs. (c, d) Electron decay dynamics of Au@$Cu_{2-x}S$ NP with excitation of 400 and 640 nm pump at (c) the wavelength of Au plasmon absorption (573 nm) and (d) the wavelength of $Cu_{2-x}S$ plasmon absorption (890 nm).

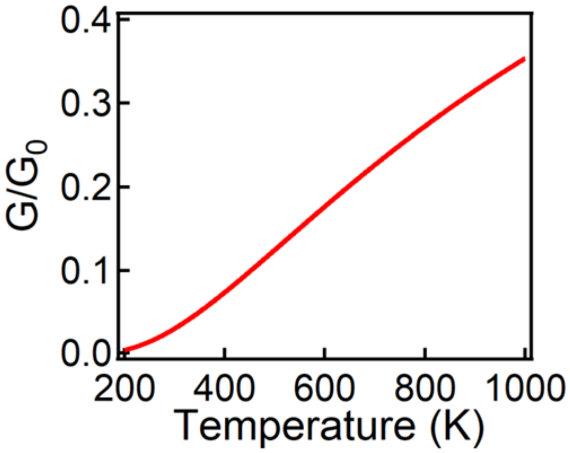

Figure S7 Change of $Cu_{2-x}S$ conductance with temperature.

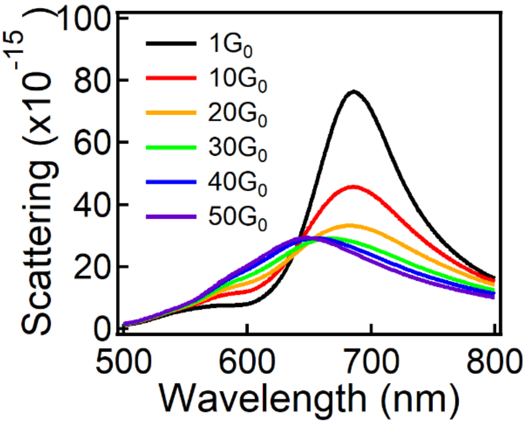

Figure S8 Simulated scattering spectra of Au@$Cu_{2-x}S$ NPoM with different gap conductance.

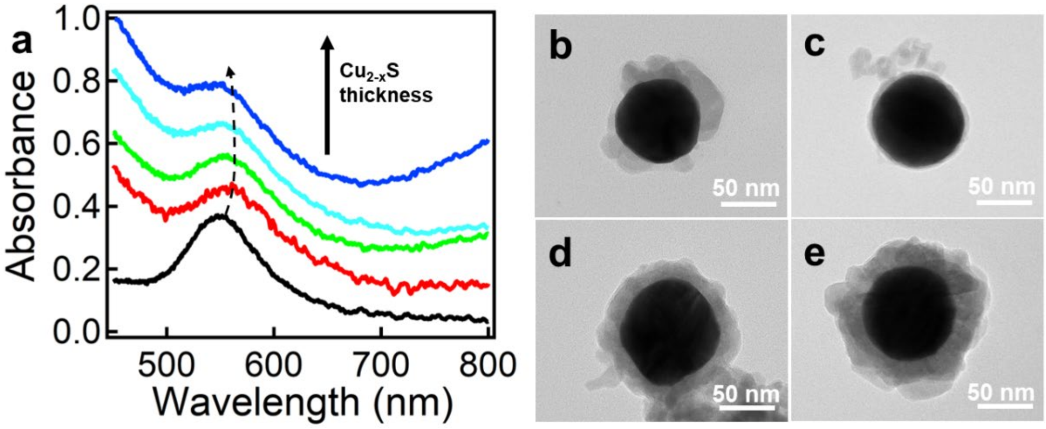

Figure S9 Chemical synthesis of Au@$Cu_{2-x}S$ NPs with variable shell thickness. (a) Extinction spectra of Au@$Cu_{2-x}S$ NPs with increased amount of $CuCl_2$ precursors added. Note the plasmon peak first redshift then slightly blueshift due to the quantum tunnelling effect as revealed previously [1]. (b-e) TEM images of the Au@$Cu_{2-x}S$ core shell NPs synthesized by adding (b) 10 μL, 20 μL, 30 μL and 40 μL of $CuCl_2$ (0.01 mM), respectively.

Reference

1. Ma S, Yang DJ, Ding SJ *et al.* Tunable Size Dependence of Quantum Plasmon of Charged Gold Nanoparticles. *Phys Rev Lett* 2021; **126**: 173902.
